# The work of the Kuru Field Unit, Kuru Research Project of the Papua New Guinea Institute of Medical Research and MRC Prion Unit

**DOI:** 10.1098/rstb.2008.4021

**Published:** 2008-11-27

**Authors:** Wandagi H. Pako

**Affiliations:** Papua New Guinea Institute of Medical ResearchPO Box 60, Goroka, EHP 441, Papua New Guinea

This is my third year working for the Kuru Research Project and I have been assigned to take charge of the fieldwork. Our fieldwork is based in Waisa village and we have nine field patrol officers who make five teams for kuru surveillance right around the four corners of Okapa District. There are two technical officers based in the field transcribing interview tapes recorded by Jerome Whitfield, Sena Anua and myself.

One patient, a man aged 62 years, died of kuru in March 2005. There were 14 suspected cases of kuru from January 2005 to September 2007:

- five suspected kuru patients have ‘recovered’ and are well;
- five suspected kuru patients are still sick with no change of symptoms and are diagnosed as not having kuru; and
- four suspected kuru patients have died from other disease.

The other responsibilities in the field are:

- Arrange field patrols for kuru surveillance of villages in remote parts of the kuru-affected region every quarter and prepare a report.
- Assist Jerome Whitfield in conducting interviews on the cultural and mortuary practices of the Atigina and Pamusagina people.
- Diagnose and film suspected kuru patients and compile reports for Jerome and Michael Alpers.
- Check and regularly report on the sickness status of suspected kuru patients.
- Transcribe recorded tapes from the Fore language into English.

